# Association between ELABELA Serum Concentrations in First Trimester and Pregnancy-Induced Hypertension

**DOI:** 10.1155/2020/2051701

**Published:** 2020-09-28

**Authors:** Shujuan Ma, Chuhao Guo, Jiayue Zhang, Sisi Long, Hongzhuan Tan, Yiping You

**Affiliations:** ^1^Department of Epidemiology and Health Statistics, Xiangya School of Public Health, Hunan Provincial Key Laboratory of Clinical Epidemiology, Central South University, Changsha, China; ^2^Department of Obstetrics, Hunan Provincial Maternal and Child Health Hospital, Changsha, China

## Abstract

ELABELA (ELA) is considered to be implicated in the pathophysiology of preeclampsia (PE), since ELA-deficient mice exhibited PE-like symptoms and infusion of exogenous ELA normalized the gestational hypertension (GH) and proteinuria. However, no evidence show that circulating ELA is deficient in early placental development among women who destined to develop GH/PE. This nested case-control study was conducted to investigate the association between serum ELA concentration in early pregnancy and the risk of later GH/PE. Participants were recruited and sampled in 10-14^+6^ weeks of gestation. Definite GH/PE cases were matched 1 : 3 to controls with respect to age and gestational age. Serum concentration of ELA was measured using enzyme immunoassay. Women with later GH (*N* = 28) had a slightly lower median concentration of ELA (46.72 ng/mL versus 53.54 ng/mL), while those with later PE (*N* = 16) had a slightly higher median concentration of ELA (74.8 ng/mL versus 66.30 ng/mL) compared to the controls. Yet, both the increments did not reach statistically significant difference (GH: *P* = 0.380, PE: *P* = 0.799). ELA serum concentrations were unchanged in first trimester in women with GH/PE. Further studies are still needed to identify the dynamic changes in serum ELA concentrations during the whole pregnancy, especially in those with pregnancy-induced hypertensive disorders.

## 1. Introduction

Pregnancy-induced hypertension (PIH), including gestational hypertension (GH) and preeclampsia (PE), complicates 5-7% of pregnancies [1] and is a major cause of perinatal and maternal morbidity and mortality [2], especially in low-income and middle-income countries [3]. Despite great efforts have been made to investigate the pathogenesis of GH and PE, the underlying mechanism has not been fully elucidated [4]. Typical physiological and pathological changes of GH and PE are abnormal vascular responses to placentation [5]. Current knowledge and hypothesis suggest that altered placental development and abnormal cytotrophoblast invasion of spiral arterioles in early pregnancy may play critical roles in the progression of events, which initiate a spectrum of pregnancy-induced hypertensive disorders [6, 7].

Recently, a new endogenous ligand of the apelin receptor (APJ), named ELABELA (ELA), has been identified and shown crucial functions in embryos and adult organisms, such as endoderm differentiation, heart morphogenesis, self-renewing of human embryonic stem cells, angiogenesis, and blood pressure control [8, 9]. Emerging evidence reveal that ELA is a pregnancy-associated hormone which can be secreted by the developing conceptus and placenta [10–13]. Ho et al. [10] generated the ELA-knockout mice through homologous recombination and found that ELA deficiency resulted in angiogenesis defective embryos and delayed syncytiotrophoblast differentiation. More interesting, ELA-deficient mice exhibited PE-like symptoms with hypertension, proteinuria, and kidney injury during pregnancy, and all of these could be rescued by exogenous recombinant ELA [10]. Similarly, ELA is predominantly expressed by trophoblasts in the chorionic villi of human placentas and significantly potentiated trophoblast invasion in vitro assays [10, 14]. Zhou et al. [15] identified ELA to significantly decrease in late-onset PE, ELA levels were significantly decreased in late-onset PE pregnancies compared with normal pregnancies, the mRNA and protein expressions of ELA and APJ in late-onset PE placental tissues were also decreased.

These studies support that ELA may be implicated in the pathophysiology of human GH and PE, reduced circulating ELA, especially in the first trimester, may lead to reduced trophoblast invasion and a dysfunctional placenta, which subsequently cause hypertension and other PE symptoms to develop in third trimester. As yet there is no evidence that circulating ELA is deficient in early placental development among those destined to develop pregnancy-induced hypertensive disorders. Thus, we conducted this study to investigate the association between serum ELA concentration in early pregnant and the risk of human later GH/PE.

## 2. Materials and Methods

This nested case-control study was based on an early pregnancy follow-up cohort of 872 single pregnancy women, which was established in Hunan Provincial Maternal and Child Health Hospital (HPMCHH) in South China, from Mar 2017 to Mar 2018 (ChiCTR1900020652). Pregnant women were recruited in 10-13^+6^ weeks of gestation. The gestational age was calculated by the outpatient doctor based on the last menstrual period and results of ultrasound examination. The inclusion criteria were as follows: (i) singleton pregnancy and natural conception; (ii) without diseases like prepregnancy diabetes, thyroid disorders, hypertension, and cardiovascular diseases; and (iii) planned to complete regular obstetric examinations and the final delivery at current hospital. All the eligible participants gave written informed consent, and the study protocol was approved on Jan 11, 2017, by the Medical Ethical Committee of HPMCHH (EC201624).

GH and PE were defined according to the 2013 American College of Obstetricians and Gynecologists criteria [16]. Controls were recruited from the same cohort with normal blood pressure during all the pregnancy and matched 3 : 1 to GH and PE cases with respect to age (±3 years) and gestational age (±1 week). Demographic characteristics were collected from validated questionnaires. Anthropometric data were collected according to standard procedures. Body mass index (BMI) was calculated by dividing the weight (kg) by the square of height (m). Systolic pressure (SBP) and diastolic pressure (DBP) were measured using a wrist electronic sphygmomanometer (Omron HEM-6050, Japan) in a sitting position after a 15-minute rest. Information about routine biochemical indicators, such as hemoglobin (HGB), triglycerides (TG), total cholesterol (TCHOL), high-density cholesterol (HDL), low-density cholesterol (LDL), alanine aminotransferase (ALT), aspartate aminotransferase (AST), and uric acid (UA), were extracted from clinical records of the Hospital Information Systems used in HPMCHH. Childbirth-related information (delivery gestational age, mode, birth weight, and fetal sex) were extracted from the birth record of each participant.

Blood samples were collected in 10-14^+6^ weeks of gestation. Serum concentration of ELA was measured using a sensitive and specific enzyme immunoassay (human ELA Elisa kit manufactured by Phoenix Pharmaceuticals Inc., lot No. 607977). This kit is designed to detect human ELA-32 and its related peptides based on the principle of “competitive” enzyme immunoassay, and the detection range is 0 to 100 ng/mL. The testing was strictly according to the manufacturer's instructions, except that extraction of peptides was not performed prior to ELISA quantification. The internal correlation coefficients of the standard curve in our experiment ranged from 0.92 to 0.94, and the range of coefficient of variation for duplicates was 0.08-14.52%. All data were analyzed by paired logistic regression model fitted by the hierarchical COX model. Statistical analyses were performed using SPSS (V 23.0); *P* < 0.05 was considered significant, and scatter plots were performed using GraphPad Prism (V 5.0).

## 3. Results

### 3.1. Characteristics of the Study Population

A total of 872 subjects were included in the early pregnancy follow-up cohort. Excluding those who were lost to follow-up, 744 were successfully followed up to 42 days postpartum, of which, 46 cases of PIH (30 GH and 16 PE) were diagnosed, and 44 cases (28 GH and 16 PE) with complete sample data were included in this study. The case numbers of early-onset (<34 weeks of gestation) and late-onset (>34 weeks of gestation) PE were 4 and 12, respectively. The demographic and clinical characteristics of cases and controls are summarized in Table 1. All the patient groups (PIH, GH, and PE) had a significant higher average prepregnant BMI, SBP, and DBP in the early pregnancy. PE patients also had significant higher average UA (*P* = 0.014), higher average TG (*P* = 0.014), and lower average HDL (*P* = 0.005) in the early pregnancy, as well as lower average birthweight (*P* = 0.004) and gestational age at delivery (*P* = 0.008) than their matched controls.

### 3.2. Serum Concentration of ELA

**Figure 1:**
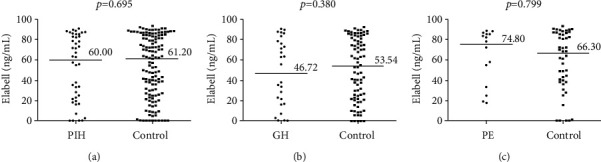
Serum concentration of ELABELA (ng/mL) in the early pregnancy (10-14 weeks of gestation) in women with later PIH (pregnancy-induced hypertension, Figure 1(a))/GH (gestational hypertension, Figure 1(b))/PE (preeclampsia, Figure 1(c)) and their 1 : 3 age- and gestational age-matched health controls. The black line represents the median. Comparisons was conducted as follows: 44 PIH cases versus 132 corresponding matched controls, 28 GH cases versus 84 corresponding matched controls, and 16 PE cases versus 48 corresponding matched controls.

The differences of circulating ELA levels in the early pregnancy between all case groups and their corresponding matched controls were not significant (PIH: *P* = 0.695, GH: *P* = 0.380, and PE: *P* = 0.799; Figure 1). PE patients had a slightly higher median concentration of ELA in the early pregnancy than their counterparts with a normal pregnancy (median: 74.80 ng/mL (IQR, 26.78-85.33 ng/mL) vs. median: 66.30 ng/mL (IQR, 38.42-85.86), Figure 1(c)). Women with late-onset PE (median: 74.8 ng/mL; IQR, 20.59-86.46 ng/mL) had a slightly higher median concentration of ELA than their matched controls (median: 63.10 ng/mL; IQR, 38.91-89.56 ng/mL) and those with early-onset PE (median: 69.73 ng/mL; IQR, 39.32-84.49 ng/mL). Yet, none of these increments reached statistically significant difference. After adjusting for above variables with significant differences between groups (including pregnant BMI, HGB, UA, TG, and HDL), by hierarchical COX model, results showed that circulating ELA levels in the early pregnancy were not statistically associated with the later risk of PIH (relative risk, RR = 0.995, 95% confidence interval, 95% CI: 0.983-1.007, *P* = 0.415), GH (RR = 0.996, 95% CI: 0.984-1.008, *P* = 0.495), or PE (RR = 1.002, 95% CI: 0.984-1.008, *P* = 1.020).

## 4. Discussion

In this prospective, confounding well-controlled study, we did not find differences of circulating ELA levels in the early pregnancy between women with later PIH/PE/GH and those with a normal pregnancy. This did not support the hypothesis that circulating ELA might be deficient in early placental development among women who destined to develop pregnancy-induced hypertensive disorders [10].

In line with our findings, four recent reports drew a similar conclusion in human. First, Villie et al. [17] investigated circulating ELA levels before PE onset (mean gestation age at sampling was about 24 weeks) and indicated that ELA concentrations did not differ between 12 PE patients and 14 controls (mean was 11.86 ± 10.8 versus 8.71 ± 7.7 ng/mL). Second, Pritchard et al. [18] found no difference of circulating ELA concentrations (gestation age at sampling ranged from 26.7 weeks to 30.9 weeks) between 32 women with preterm PE and 32 matched controls (median, 28.5 pg/mL; 95% CI, 5.3 to 63.2 versus median, 20.5 pg/mL; 95% CI, 9.2 to 58.0, respectively); moreover, placental mRNA (encoding ELA) was unchanged in 82 PE patients compared with 82 matched healthy controls (mean difference, 0.53%; 95% CI, -25.9 to 27.0; *P* = 0.78). Third, Panaitescu et al. [19] did not find the differences in ELA plasma concentrations (gestation age at sampling ranged from 27.4 weeks to 32.0 weeks) between women with early-onset PE (6.09 ng/mL, IQR: 2.8-10.66 ng/mL) and their counterparts with a normal pregnancy (median: 4.02 ng/mL, IQR: 3.26-7.49 ng/mL). Last, Huang et al. [20] also suggested that the ELA levels were similar between PE women and normotensive controls throughout pregnancy. In summary, although these studies have different study design, sample size, sampling gestational age, and ELA concentration detection range, they all seem to indicate the same conclusion that ELA is not deficient in PE patients either before or after the onset. ELA levels in circulation might be an unlikely candidate to function as a first trimester preeclampsia screening biomarker [21]. Despite our effects to advance the sampling time to early pregnant, several limitations in our study needed to be addressed and merited further discussion. First, the sample size was small, especially the PE group. Second, all of the participants were from the same research site, and blood samples collected at a single time point would not convey the entire dynamic relationship. Third, we did not perform sample extraction prior to ELISA quantification as recommended, which might lead to ambiguous finding [22].

Two ways of ELA were proposed to prevent the hypertension in pregnant mice: curbed inappropriate differentiation of endothelial tips cells and modulated maternal cardiorenal function [10]. The PE-protective effects of ELA in the ELA-deficient mice were presumably achieved through APJ signaling in the endothelial cells, but other possible contribution of unidentified ELA receptors could not be precluded [23]; especially in other species, like humans, the effects may not even stand out. Moreover, maternal circulating ELA comes from both the placenta and human organs, like the kidney and heart [24, 25], and then the potential compensation and feedback mechanisms may also differ from ELA-knockout rodents. A detailed mechanism of the difference between the results from mice and human is still unclear, and more well-designed large sample size studies are needed to identify the dynamic changes in serum ELA concentration during the human's whole pregnancy, especially in those with PIH-complicated pregnancies.

## Figures and Tables

**Table 1:** Demographic and clinical characteristics of the study population.

	All the controls	PIH		GH		PE	
	*N* = 132	*N* = 44	*P* value^†^	*N* = 28	*P* value^†^	*N* = 16	*P* value^†^
Demographic characteristics							
Age	31.12 ± 4.02	31.05 ± 3.92	0.645	29.68 ± 3.32	0.399	33.44 ± 3.81	0.627
Prepregnant BMI	20.63 ± 2.16	22.47 ± 3.37	﹤0.001	21.42 ± 2.87	0.034	24.3 ± 3.46	0.005
Gravidity	2.25 ± 1.19	2.07 ± 1.23	0.289	1.75 ± 0.84	0.210	2.63 ± 1.59	0.857
Parity	0.54 ± 0.54	0.50 ± 0.55	0.662	0.46 ± 0.51	1.000	0.56 ± 0.63	0.426
PIH history	0 (0/132)	9.1% (4/44)	0.316	7.1% (2/28)	0.616	12.5% (2/16)	0.478
Smoking history	7.6% (10/132)	6.8% (3/44)	0.873	7.1% (2/28)	0.640	6.3% (1/16)	0.521
Drink history	4.5% (6/132)	2.3% (1/44)	0.521	0 (0/28)	0.619	6.3% (1/16)	0.797
Clinical characteristics in early pregnancy							
Gestational age at sampling (weeks)	12.70 ± 0.77	12.68 ± 0.90	0.752	12.78 ± 0.82	0.407	12.49 ± 1.02	0.250
SBP (mmHg)	109.38 ± 6.15	122.75 ± 9.98	<0.001	121.68 ± 10.75	<0.001	124.63 ± 8.46	0.002
DBP (mmHg)	70.18 ± 5.60	79.98 ± 7.32	<0.001	79.21 ± 7.26	<0.001	81.31 ± 7.45	0.002
HGB (g/L)	123.53 ± 7.64	126.61 ± 7.82	0.026	126.79 ± 7.57	0.120	126.31 ± 8.47	0.091
ALT (U/L)	19.36 ± 17.07	22.72 ± 16.04	0.224	20.46 ± 16.12	0.288	26.69 ± 15.61	0.494
AST (U/L)	19.45 ± 7.97	21.27 ± 8.24	0.187	19.92 ± 6.98	0.510	23.63 ± 9.89	0.219
UA (*μ*mol/L)	199.33 ± 42.31	216.98 ± 52.46	0.029	203.39 ± 46.01	0.672	240.75 ± 55.96	0.014
TG (mmol/L)	1.46 ± 0.51	1.73 ± 0.77	0.021	1.55 ± 0.65	0.682	2.04 ± 0.88	0.014
TCHOL (mmol/L)	4.68 ± 0.69	4.57 ± 0.67	0.335	4.67 ± 0.74	0.942	4.40 ± 0.51	0.145
HDL (mmol/L)	2.04 ± 0.42	1.87 ± 0.48	0.032	2.00 ± 0.47	0.980	1.64 ± 0.40	0.005
LDL (mmol/L)	2.49 ± 0.66	2.49 ± 0.57	0.994	2.50 ± 0.66	0.709	2.47 ± 0.38	0.839
Clinical characteristics after onset							
GDM^‡^	14.4% (19/132)	20.5% (9/44)	0.342	14.3% (4/28)	0.746	31.3% (5/16)	0.204
Highest SBP before labor (mmHg)	117.60 ± 6.90	150.00 ± 13.00	<0.001	145.86 ± 5.95	0.002	157.25 ± 18.23	0.015
Highest DBP before labor (mmHg)	76.77 ± 5.00	97.91 ± 8.84	<0.001	94.82 ± 4.83	0.001	103.31 ± 11.55	0.013
Gestational age at delivery (weeks)	39.50 ± 1.08	38.86 ± 1.85	0.006	39.58 ± 1.17	0.790	37.61 ± 2.17	0.008
Birthweight (g)	3388.48 ± 368.70	3119.09 ± 625.83	0.002	3362.5 ± 375.31	0.406	2693.13 ± 751.72	0.004
Delivery			0.140		0.825		0.015
Vaginal	51.5% (68/132)	38.6% (17/44)		57.1% (16/28)		6.3% (1/16)	
Cesarean section	48.5% (64/132)	61.4% (27/44)		42.9% (12/28)		93.8% (15/16)	
Fetal sex			0.426		0.647		0.773
Male	45.5% (60/132)	52.3% (23/44)		53.6% (15/28)		50.0% (8/16)	
Female	54.5% (72/132)	47.7% (21/44)		46.3% (13/28)		50.0% (8/16)	

^†^Comparison was conducted between the designated patient group and their 1 : 3 age- and gestational age-matched health controls: 44 PIH cases versus 132 corresponding matched controls, 28 GH cases versus 84 corresponding matched controls, 16 PE cases versus 48 corresponding matched controls. ^‡^Gestational diabetes mellitus (GDM) was defined using the criteria from the International Association of Diabetes and Pregnancy Study Groups based on the results of a standard 2-hour 75 g oral glucose tolerance test at 24-28 weeks of gestation. ALT; alanine aminotransferase; AST: aspartate aminotransferase; BMI: body mass index; DBP: diastolic pressure; GDM: gestational diabetes mellitus; GH: gestational hypertension; HDL: high-density cholesterol; HGB: hemoglobin; LDL: low-density cholesterol; PE: preeclampsia; PIH: pregnancy-induced hypertension; SBP: systolic pressure; TCHOL: total cholesterol; TG: triglycerides; UA: uric acid.

## Data Availability

The data used to support the findings of this study are available from the corresponding author upon request.
